# Electroacupuncture for postoperative pain in mixed hemorrhoids: A meta-analysis

**DOI:** 10.1097/MD.0000000000032247

**Published:** 2022-12-16

**Authors:** Binglin Du, Zhongmiao Xu, Xin Zhong

**Affiliations:** a Department of Anorectal, South District of Guang, Anmen Hospital, Academy of Chinese Medical Sciences, Beijing, PR China; b Department of Pharmacy, Fangzhuang Community Health Service Center, Beijing, PR China; c Department of Mastopathy, Dongfang Hospital of Beijing University of Chinese Medicine, Beijing, PR China.

**Keywords:** electroacupuncture, mixed hemorrhoids, postoperative pain

## Abstract

To investigate the efficacy of electroacupuncture for postoperative pain in mixed hemorrhoids.

**Methods:**

Randomized controlled trials were searched in PubMed and Cochrane Library. The risk of bias assessment tool was used to assess methodological quality. Stata 14.0 software was used for meta-analysis. Weighted mean differences were calculated if all outcome variables were reported the same way, while standardized mean differences (SMD) were calculated if they were different.

**Results:**

From 27 identified studies, 5 Chinese studies (465 patients) were included in this meta-analysis. The electroacupuncture group had significantly lower postoperative pain scores compared with the control group at 6 hours postoperatively (SMD = –0.89, 95% CI: –1.091 to –0.692; *P* < .001), at 12 hours postoperatively (SMD = –1.089, 95% CI: –1.336 to –0.843; *P* < .001), at 24 hours postoperatively (SMD = –0.548, 95% CI: –0.721 to –0.374; *P* = .547), and 72 hours postoperatively (SMD = –1.089, 95% CI: –1.336 to –0.843; *P* < .001).

**Conclusion:**

Electroacupuncture can improve pain after surgery for mixed hemorrhoids. It is an effective method to improve the pain after hemorrhoidectomy, which deserves further research and promotion.

## 1. Introduction

Postoperative pain after hemorrhoidectomy for mixed hemorrhoids affects patient prognosis. The main causes of pain in postoperative patients are related to surgical manipulations, such as tissue excision, ligation, local compression, et cetera, that directly damage the tissues, leading to inflammatory reactions and pain.^[1,2]^ In addition, the perianal region is rich in nerve endings.^[3,4]^ Severe postoperative pain decreases the prognosis and quality of life and can cause serious psychological damage to the patients.^[5]^ Therefore, effective postoperative analgesia is the key to improving patients’ quality of life and prognosis. Enhanced recovery after surgery is effective and comprehensive for reducing the surgery recovery time and includes various components such as early ambulation, pain control, early enteral nutrition, and minimally invasive surgery.^[6,7]^

Acupuncture has a long history of treating pain. Acupuncture is 1 of the most popular treatment modalities of traditional Chinese medicine and has been used for more than 3000 years in the east Asian region, combined with herbal medicine and tuina therapy, and it is known to activate the nervous systems.^[8]^ Nevertheless, clinical studies on the efficacy of acupuncture are lacking due to the difficulty of implementing double-blind trials and the complicated assessment of the effectiveness of acupuncture. Positive controls are more relevant for clinicians in clinical acupuncture studies than blank controls.^[9]^ Yuan et al systematically evaluated the literature and concluded that acupuncture is more effective than blank controls, but no studies have been done on positive controls.^[10]^

The Yellow Emperor’s Classic of Internal Medicine clearly stated the efficacy of acupuncture.^[11]^ In recent years, modern medical practitioners have used scientific methods to innovate continuously, combining traditional acupuncture with modern electric current technology to form a new method effective in treating postoperative pain.^[12,13]^ Electroacupuncture has shown good effects in many postoperative analgesic fields, such as pain after thoracotomy, abdominal pain after intestinal obstruction, pain after total hip replacement, pain after joint replacement, et cetera.^[14–18]^ This meta-analysis will investigate the efficacy of electroacupuncture for postoperative pain in mixed hemorrhoids.

## 2. Method

### 2.1. Literature search

Randomized controlled trials (RCTs) comparing electroacupuncture for postoperative pain in mixed hemorrhoids and evaluating its efficacy on pain relief were searched in PubMed and Cochrane Library from database inception up to December 2021. The literature search was conducted using the following English terms: acusector, electropuncture, hemorrhoid, hemorrhoidectomy, postoperative pain, and randomized controlled trials. The references cited by the relevant studies were searched manually.

### 2.2. Inclusion and exclusion criteria

The inclusion criteria were as follows: RCT, with no restriction regarding blinding; Patients underwent open (Milligan Morgan or external peel and tie) or closed hemorrhoidectomy (Ferguson hemorrhoidectomy); Patients in the electroacupuncture group received electroacupuncture or/and electroacupuncture combination treatment before or after surgery; Postoperative pain was assessed within 1 week and/or 2 to 4 weeks after hemorrhoidectomy.

The exclusion criteria were as follows: Non-randomized controlled clinical trials or RCT with incorrect randomization methods; Unclear or no diagnostic criteria or diagnostic criteria inconsistent with mixed hemorrhoids; The experimental group did not include acupuncture the experimental and control groups were a comparison of the efficacy of 2 acupuncture therapies; Multiple submissions or duplicate publications; Animal, cell, or histological studies.

### 2.3. Data extraction

Data from the included studies were extracted independently by 2 investigators. The data included baseline characteristics of the literature (author, year of publication, and country of trial), case information (sample size of trial and control groups, and type of surgery), and outcome assessment characteristics (time point of postoperative pain assessment).

### 2.4. Literature quality assessment

The risk of bias assessment tool recommended by the Cochrane Handbook for Systematic Reviews of Interventions v5.1.0 was used to assess the methodological quality of the studies.^[19]^ The assessment included randomization methods, allocation concealment methods, implementation of blinding, completeness of outcome data, selective reporting of study results, and other sources of bias. All items were rated as “yes,” “no,” and “unclear.” The assessment was performed independently by 2 investigators.

### 2.5. Statistical analysis

The meta-analysis was performed using the Stata 14.0 software. Postoperative pain scores were used as outcome variables, and weighted mean differences were calculated if all outcome variables were reported in the same way, while standardized mean differences (SMD) were calculated if they were different. Due to some clinical heterogeneity among the included studies, the effect indicators were combined using the random-effects model. The test for publication bias was completed by observing the symmetry of the funnel plot and by Begg’s test and Egger’s test.

### 2.6. Ethics approval and consent to participate

This article is a meta-analysis. The data comes from published articles and does not require ethical approval.

## 3. Results

### 3.1. Literature search screening and inclusion and bias evaluation

**Figure 1.**
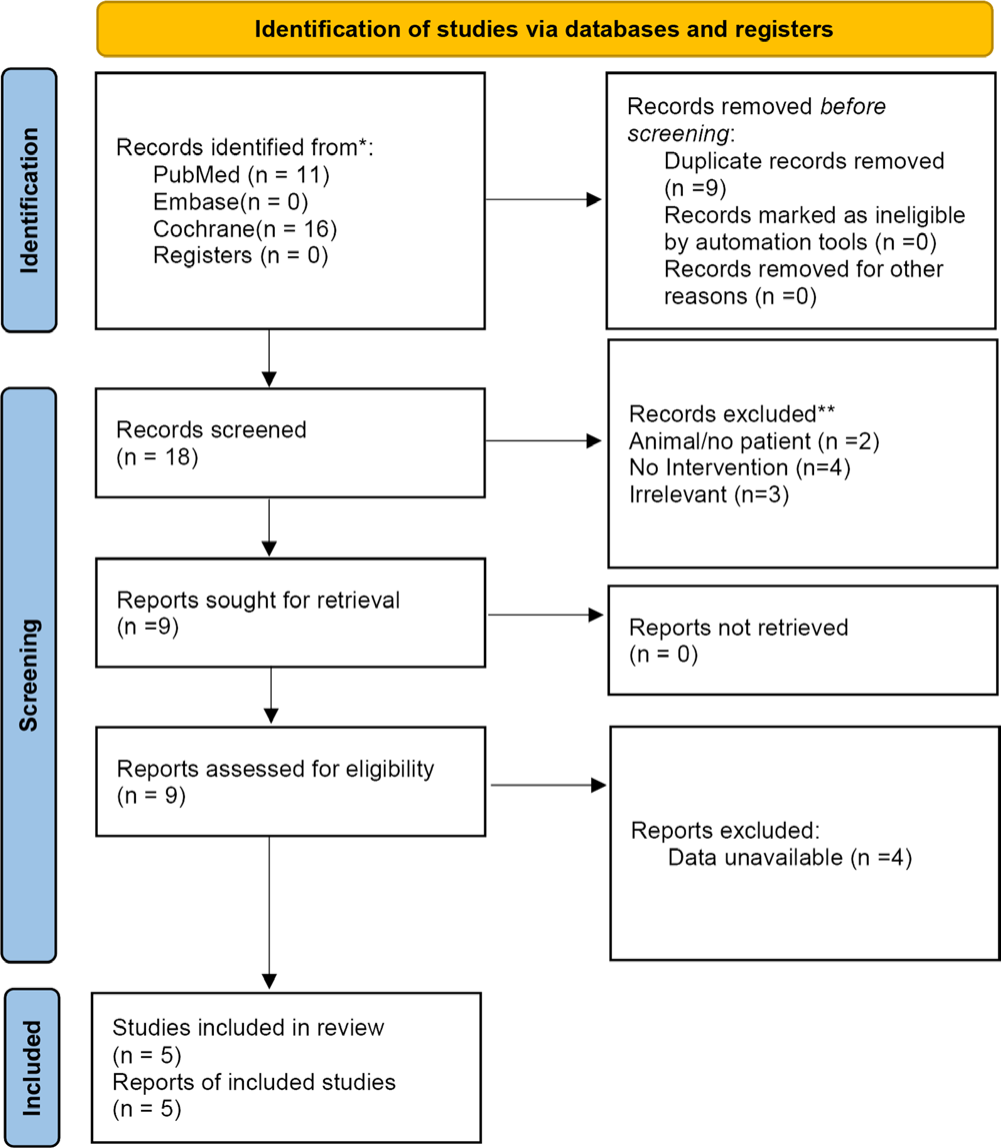
Flow chart for inclusion in the literature.

Twenty-seven records were identified. After removing 9 duplicates, 18 records were screened. Two were excluded for animal or no patient study, 4 for no interventions, and 3 as irrelevant. Nine reports were retrieved and assessed for eligibility. Four reports had no available data and were excluded. Therefore, 5 Chinese RCTs^[20–24]^ were included in this meta-analysis, including 465 patients: 270 in the electroacupuncture or combined electroacupuncture group and 195 in the control group. The characteristics of the included studies are shown in Table 1. The quality evaluation indexes of the included RCTs were satisfied, except that none of them used blinding, and the overall bias of the included literature was small because the effect of whether blinding was used on the results was relatively small. Table 2 shows the quality of the included studies. Figure 1 shows the literature search process.

**Table 1:** Characteristics of the included studies.

Author	Year	Study design	Patients	Diagnostic criteria	Surgical technique	Validated scale	Anesthesic method	Regular treatment	Acupuncture treatment	Device information	Control treatment	Scoring time	Num Acu	Num Con
Song YY^[1]^	2019	prospective	June, 2016 to December, 2017 Department of Anorectal Unit, Second Chinese Hospital of Jiangsu Province	Guidelines for the Treatment of Common Diseases in Chinese Medicine and Proctology ^[2]^.	Hybrid hemorrhoid anastomosis with suprahemorrhoidal circumferential hemorrhoidectomy and stapling (PPH procedure) according to Provisional specification for suprahemorrhoidal circumferential hemorrhoidectomy and stapling ^[3]^	Referring to the guideline of clinical research of new Chinese medicine ^[4]^, combined with the clinical The postoperative observation indexes and scoring criteria of mixed hemorrhoids were formulated in the clinic	Anesthesia at the lumbar point	After 6 h of fasting, the patients were fed a liquid diet, given regular low-flow oxygen for 6 h, and monitored with electrocardiogram for 6 h. The patients were treated with regular rehydration, anti-inflammatory and hemostatic therapy. All patients were All patients were routinely changed at 16 h postoperatively	electroacupuncture:Baliao point	SDZ-V electronic needle therapy instrument, dense and sparse wave, frequency 2 Hz/15 Hz	Operation only	6h,12h,18h,24 h Postoperatively	30	30
Wen Y ^[5]^	2017	prospective	January, 2014 to January, 2016 Department of Traditional Chinese Medicine, Southwest Medical University Hospital	Guide to the Clinical Management of Hemorrhoids (2006 Edition) ^[6]^	External stripping and internal tying Tie surgery	visual analogue scale (VAS)	Anesthesia at the lumbar point	All patients underwent soap and water enema for 6 h before surgery, no water fasting, prophylactic antibiotics for 48 h after surgery, hemostatic drugs for 3 d, liquid diet for 2 d, defecation for 2 d after surgery, regular defecation with Chinese medicine, warm saline sitz bath for 5-10 min after defecation, and local drug change.	Electroacupuncture and electroacupuncture combined with buried thread:Chengshan, Changqiang point	SDZ-V electronic acupuncture therapy instrument, dense and sparse wave	Buried thread	4h,12h,24h,72h,7d Postoperatively	40	40
Long Q^[7]^	2018	prospective	May, 2016 to May, 2017 Department of Traditional Chinese Medicine, Affiliated Hospital of Southwest Medical University	Guidelines for the Treatment of Common Diseases in Chinese Medicine and Proctology ^[2]^.	External stripping and internal tying Tie surgery	VAS	Anesthesia at the lumbar point	All patients were admitted to the hospital for preoperative education, perfect preoperative related examination, and enema 4 h before surgery. Postoperatively, all patients were given routine antibiotics to prevent infection, hemostasis and symptomatic treatment such as drug changes.	Electroacupuncture and electroacupuncture combined with Ear acupressure group: Xialiao,Changqiang point	G6805-1 electronic acupuncture therapy instrument, dense and sparse wave	Ear acupressure group	4h,12h,24h,48h,72 h Postoperatively	30	30
Wu J^[8]^	2017	prospective	June, 2012 to January, 2013 West China Hospital of Sichuan University, Department of Integrative Medicine, Chinese Medicine and Fistula Specialist Unit	Chinese Medicine Industry Standard of the People’s Republic of China “Diagnostic Efficacy Criteria for Chinese Medical Evidence” (ZY/T001-94) ^[9]^	External stripping and internal tying Tie surgery	VAS	Anesthesia at the lumbar point	All patients were treated with routine antibiotics to prevent infection, stop bleeding and change medication, etc. They were given a 6-h preoperative fast, and a 40-mL enema 2 h before surgery. After the operation, all subjects were fed a liquid diet after 2h of fasting, and were treated with regular rehydration, anti-inflammation, hemostasis and other symptomatic support. All subjects resumed normal diet and regular bowel movements on the second day after surgery, and were given a fumigating sitz bath with traditional Chinese medicine after the bowel movement.	Electroacupuncture:Chengshan, Changqiang point	SDZ-V electro-acupuncture instrument, dense and sparse wave, frequency 2 Hz/ 15 Hz	Operation only	24h Postoperatively	40	40
Sun PL^[10]^	2011	prospective	January, 2008-April, 2010 Inpatient Department of Anorectology, The First Affiliated Hospital of Guangxi College of Traditional Chinese Medicine	Guide to the Clinical Management of Hemorrhoids (2006 Edition)	External stripping and internal tying Tie surgery	VAS	Local perianal nerve Anesthesia	The subjects were given anti-infection and hemostatic treatment after surgery, and received warm water sitz bath, T DP infrared light therapy, and clean change of medication after stool from the first day after surgery.	Electroacupuncture: Chengshan point	Han’s electro-acupuncture instrument (L H-202-H) takes the sparse and dense wave current intensity of 6–10 mA at 2 Hz/100 Hz	Naproxen	5h,24h,48h,72 h Postoperatively	60	55

VAS = visual analog scale.

References

[1]Song YY, Ni GX. [Effect of preoperative intervention of electroacupuncture at Baliao point on postoperative complications of procedure for prolapsed and hemorrhoids]. Zhongguo Zhen Jiu. 2019;39:253–256.

[2]Zhang Y, Han B, Tian Z. Guidelines for the treatment of common diseases in Chinese medicine anorectology. Beijing: China Traditional Chinese Medicine Press; 2012.

[3]Surgery CSoM. Anorectal Surgery group. Revision of the provisional specification of suprahemorrhoidal circumferential hemorrhoidectomy (PPH) Chin J Gastroin Surgery. 2005;8:342.

[4]Zheng X. Clinical research guidelines for new Chinese medicines. Beijing: China Medical Science and Technology Press; 2002.

[5]Wen Y, Li J, Long Q et al: [Electroacupuncture combined with catgut implantation for postoperative pain of mixed hemorrhoids]. Zhongguo Zhen Jiu. 2017;37:243–246.

[6]Surgery CSoM. Chinese society of traditional Chinese medicine, Chinese society of integrative medicine. Guidelines for the clinical management of hemorrhoids (2006 edition). Chin J Gastroin Surgery. 2006;9:46.

[7]Long Q, Li Y, Li J,et al [Clinical observation of electroacupuncture combined with auricular point sticking therapy for anal pain of mixed hemorrhoid after external excision and internal ligation]. Zhongguo Zhen Jiu. 2018;38:580–585.

[8]Wu J, Zhao Y, Yang CM et al: [Effects of electroacupuncture preemptive intervention on postoperative pain of mixed hemorrhoids]. Zhongguo Zhen Jiu. 2014;34:279–283.

[9]Medicine MSotSAoTC. 22 specialties and 95 diseases of Chinese medicine treatment plan. Beijing: China Chinese Medicine Publishing House; 2010.

[10]Sun PL, Yang W, Zhang LC. [Effect of electroacupuncture at Chengshan (BL 57) on postoperative pain of mixed hemorrhoids]. Zhongguo Zhen Jiu. 2011;31:413–415.

**Table 2:** Quality evaluation for Cochrane tool.

Study	Random sequence generation (selection bias)	Allocation concealment (selection bias)	Blinding of participants and peraonnel (performance bias)	Blinding of outcome assessmnet (detection bias)	Incomplete outcome data (attrition bias)	Selective reporting (reporting bias)	Other bias	Total quality scores
Song YY	*	*			*	*	*	5
Long Q	*	*			*	*	*	5
Wu J	*	*			*	*	*	5
Sun PL	*	*			*	*	*	5
Wen Y	*	*			*	*	*	5

### 3.2. Postoperative pain evaluation

#### 3.2.1. 6 Hours postoperatively.

**Figure 2.**
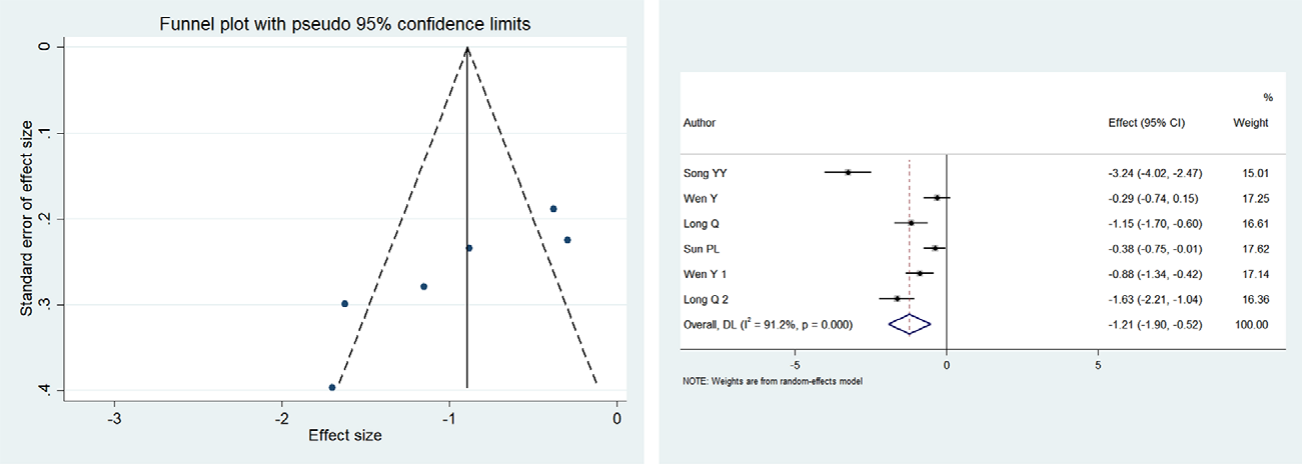
SMD of 2 groups after 6 hours postoperatively. Electroacupuncture group had significantly lower postoperative pain scores compared with the control group (SMD = - 0.89, 95% CI: -1.091– -0.692; *P* = .000). SMD = standardized mean difference.

Of the included studies, 4 reported the visual analog scale (VAS) scores at 6 hours postoperatively, with heterogeneity among studies (*I*^2^ = 91.2%, *P* < .001). The meta-analysis using the random-effects model showed that patients in the electroacupuncture group had significantly lower postoperative pain scores compared with the control group (SMD = –0.89, 95% CI: –1.091 to –0.692; *P* < .001). The funnel plot was symmetrical, with *P* > .1 for Begg’s test and *P* > .1 for Egger’s test, suggesting no publication bias. Figure 2 shows the results.

#### 3.2.2. 12 Hours postoperatively.

**Figure 3.**
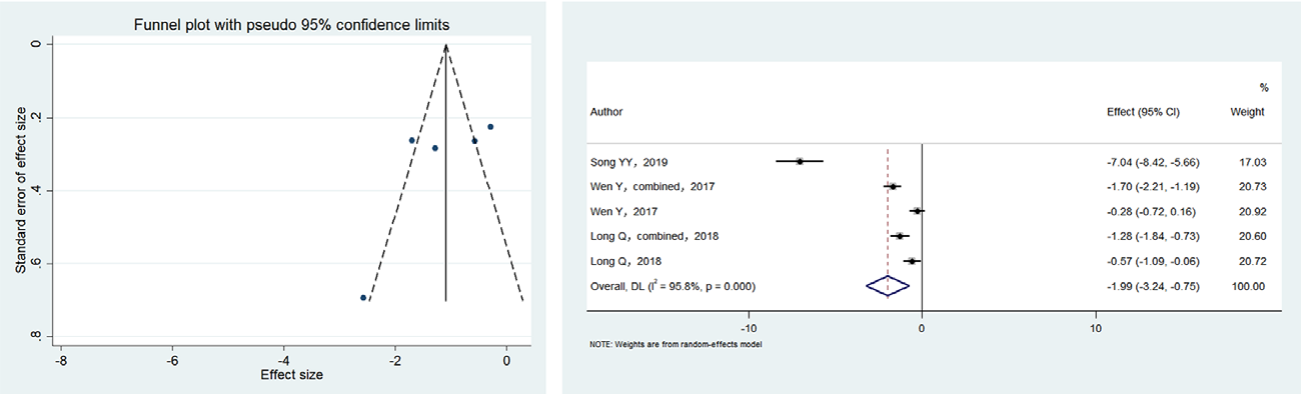
SMD of 2 groups after 12 hours postoperatively. Electroacupuncture group had significantly lower postoperative pain scores compared with the control group (SMD = -1.089 95% CI: -1.336– -0.843; *P* = .000). SMD = standardized mean difference.

Of the included studies, 3 reported the VAS scores at 12 hours postoperatively, with heterogeneity among studies (*I*^2^ = 95.8%, *P* < .001). The meta-analysis using the random-effects model showed that the patients in the electroacupuncture group had significantly lower postoperative pain scores than the control group (SMD = –1.089, 95% CI: –1.336 to –0.843; *P* < .001). The funnel plot was symmetrical, with *P* > .1 for Begg’s test and *P* > .1 for Egger’s test, suggesting no publication bias. Figure 3 shows the results.

#### 3.2.3. 24 Hours postoperatively.

**Figure 4.**
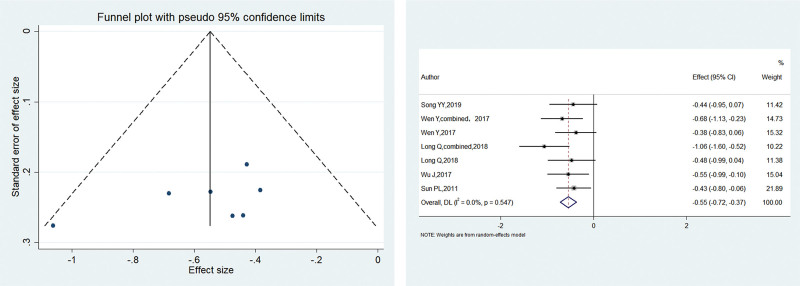
SMD of 2 groups after 24 hours postoperatively. Electroacupuncture group had significantly lower postoperative pain scores compared with the control group (SMD = -0.548 95% CI: -0.721– -0.374; *P* = .547). SMD = standardized mean difference.

All 5 included studies reported the VAS scores at 24 hours postoperatively, with no heterogeneity between studies (*I*^2^ = 0.00%, *P* < .001). The meta-analysis using the fixed-effects model showed that the patients in the electroacupuncture group had significantly lower postoperative pain scores than the control group (SMD = –0.548, 95% CI: –0.721 to –0.374; *P* = .547). The funnel plot was symmetrical, with *P* > .1 for Begg’s test and *P* > .1 for Egger’s test, suggesting no publication bias. Figure 4 shows the results.

#### 3.2.4. 72 Hours postoperatively.

**Figure 5.**
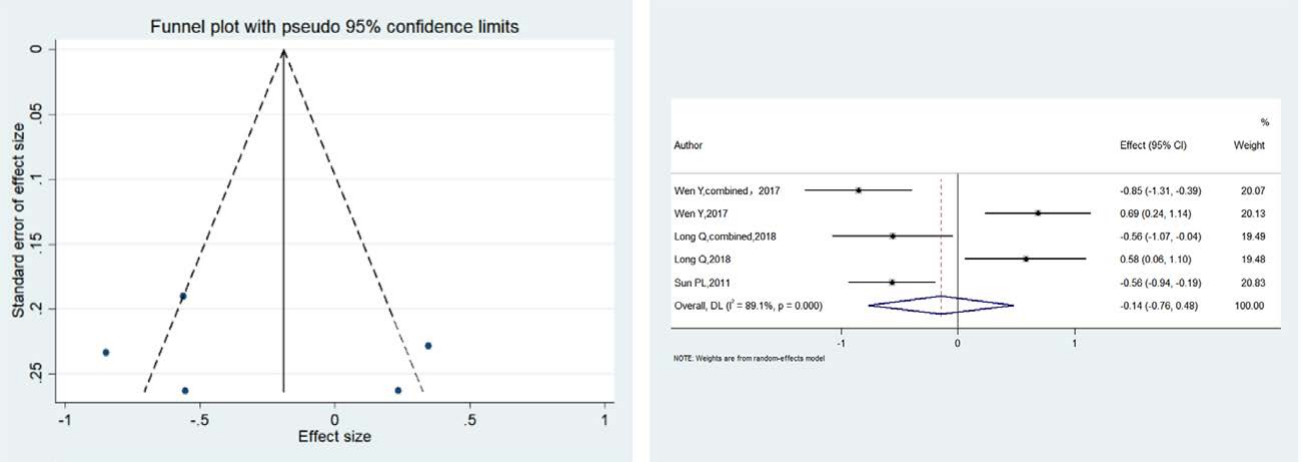
SMD of 2 groups after 72 hours postoperatively. Patients in the electroacupuncture group had significantly lower postoperative pain scores compared with the control group (SMD = -1.089 95% CI: -1.336– -0.843; *P* = .000). SMD = standardized mean difference.

Three studies reported VAS at 72 hours postoperatively, with heterogeneity among studies (*I*^2^ = 89.16%, *P* < .001). The meta-analysis using the random-effects model showed that the patients in the electroacupuncture group had significantly lower postoperative pain scores compared with the control group (SMD = –1.089, 95% CI: –1.336 to –0.843; *P* < .001), The funnel plot was symmetrical, with *P* > .1 for Begg’s test and *P* > .1 for Egger’s test, suggesting that there was no publication bias. Figure 5 shows the results.

## 4. Discussion

The main method of analgesia is to block the transmission path of injurious stimuli and increase the body’s pain threshold, using, for example, oral or intramuscular analgesic drugs, local injection of long-acting analgesics, epidural injection analgesia, self-administered analgesia, over-the-top analgesia, balance analgesia, et cetera. Nevertheless, there are disadvantages such as large adverse reactions, being expensive, and being addictive, and there is no recognized best solution. In recent years, standardized pain management has been strongly advocated internationally, with the basic principles of effective pain relief and minimizing the adverse effects of drugs and treatment costs.^[25]^ Acupuncture, as an important part of traditional Chinese medicine, has the advantages of simplicity, convenience, and experimentation and is widely used for postoperative pain in mixed hemorrhoids.^[26,27]^

This study showed that electroacupuncture significantly alleviated the pain of patients after mixed hemorrhoidectomy, and the patient’s pain scores were significantly lower at 6, 12, 24, and 72 hours after hemorrhoidectomy compared with the control group. The commonly used acupuncture points included in the included studies were Chengshan, Baliao, Changqiang, and Xiaoliao. From the anatomical structure, the Ciliao, Zhongliao, and Xialiao points are located at the exit of the sacral nerve, which contains 4 nerve fibers with different functions: parasympathetic afferent and efferent fibers, and motor and sensory fibers of the somatic nerve.^[28]^ The afferent and efferent fibers of the somatic and visceral nerves are closely connected to the brain and spinal cord through the central and peripheral nervous systems, so by stimulating the sacral nerve, the complex neural connections can be used to exert positive and extensive neurohumoral regulation on the organs in the pelvis.^[29]^ Therefore, from the perspective of traditional Chinese medicine, electroacupuncture of the Baliao points can unblock the meridians, regulate the Qi, and move blood to dissipate stasis. Electroacupuncture of the Baliao points can not only reduce the pressure in the anus, rectum, and pelvic floor and correct the dysfunction of the pelvic floor muscle synergy, but also reduce the inhibitory effect of the parasympathetic nerve, regulate the function of the bladder and urethra, and reduce postoperative complications.^[30]^ The Chengshan point is important in treating hemorrhoid disease in ancient records and fusions have mentioned that the acupuncture prescription of Chengshan in combination with Changqiang is a typical prescription of distant and near matching points.^[26]^ Chengshan point has the efficacy of opening and activating the meridians, warming the meridians, transforming dampness, and strengthening the spleen and kidney.^[31,32]^

The strengths of this study include: the included literature were all randomized controlled trials, which have a certain degree of credibility; and the literature search is more comprehensive, and no language restrictions were set, which reduces the occurrence of selection bias and makes the results more reliable. The limitations of this study include: the number of studies being too small to deepen the analysis of the acupuncture points taken, the number of treatments, and the duration of needle retention; The analysis of mixed hemorrhoids with different conditions was not done, and in the future, with the increasing number of related studies, the above deficiencies can be analyzed and explained in relevant updates; Negative studies on the subject could not be identified. Of course, publishing negative results is not easy, and the subject may suffer from a publication bias, even though the funnel plots and Begg’s and Eggers’s did not detect such bias, the results must be considered unreliable when there are < 10 studies.^[33]^

In conclusion, based on current evidence, it is believed that electroacupuncture can improve the pain of patients with mixed hemorrhoids after surgery and is an effective method to improve the pain after hemorrhoidectomy, which deserves further research and promotion.

## Author contributions

**Data curation:** Binglin Du.

**Formal analysis:** Binglin Du.

**Investigation:** Binglin Du.

**Methodology:** Xin Zhong.

**Resources:** Zhongmiao Xu.

**Writing – original draft:** Binglin Du.

**Writing – review & editing:** Xin Zhong.

## References

[1] Sheng H. Research progress of acupuncture point therapy intervention for postoperative pain of mixed hemorrhoids. N Chin Med. 2021;53:109–12.

[2] Ye M Tang Y An M. Meta-analysis of the efficacy and safety of acupuncture point buried thread to reduce postoperative pain of mixed hemorrhoids Meta-analysis of the efficacy and safety of acupuncture points to reduce postoperative pain of mixed hemorrhoids. Chin Med Bull. 2019;18:44–50.

[3] Rogers J. Testing for and the role of anal and rectal sensation. Baillieres Clin Gastroenterol. 1992;6:179–91. 1586768 10.1016/0950-3528(92)90026-b

[4] Li L Li Z Hou HS. Sensory nerve endings in puborectalis and anal region: normal findings in the newborn and changes in anorectal anomalies. J Pediatr Surg. 1990;25:658–64. 2141634 10.1016/0022-3468(90)90357-f

[5] Gan D Ji F Cui C. Combination of intravenous laryngeal mask anesthesia with pubic nerve block anesthesia in mixed hemorrhoid surgery. Colorectal Surg Sec. 2020;26:687–90.

[6] Barbieux J Hamy A Talbot MF. Does enhanced recovery reduce postoperative ileus after colorectal surgery? J Visc Surg. 2017;154:79–85. 27618698 10.1016/j.jviscsurg.2016.08.003

[7] Feng F Ji G Li JP. Fast-track surgery could improve postoperative recovery in radical total gastrectomy patients. World J Gastroenterol. 2013;19:3642–8. 23801867 10.3748/wjg.v19.i23.3642 PMC3691044

[8] Coutaux A. Non-pharmacological treatments for pain relief: TENS and acupuncture. Joint Bone Spine. 2017;84:657–61. 28219657 10.1016/j.jbspin.2017.02.005

[9] Guideline IHT. Choice of control group and related issues in clinical trials E10. Choice 2000. 2000.

[10] Yuan QL Guo TM Liu L. Traditional Chinese medicine for neck pain and low back pain: a systematic review and meta-analysis. PLoS One. 2015;10:e0117146. 25710765 10.1371/journal.pone.0117146 PMC4339195

[11] Curran J. The yellow emperor’s classic of internal medicine. Brit Med J. 2008;336:777.

[12] Long Q Li Y Li J. [Effect of electroacupuncture preconditioning with different frequencies on anal pain after milligan-morgan hemorrhoidectomy]. Zhongguo Zhen Jiu. 2019;39:477–81. 31099217 10.13703/j.0255-2930.2019.05.005

[13] Langenbach MR Aydemir-Dogruyol K Issel R. Randomized sham-controlled trial of acupuncture for postoperative pain control after stapled haemorrhoidopexy. Colorectal Dis. 2012;14:e486–91. 22330010 10.1111/j.1463-1318.2012.02984.x

[14] Park S Lyu YR Park SJ. Electroacupuncture for post-thoracotomy pain: a systematic review and meta-analysis. PLoS One. 2021;16:e0254093. 34234358 10.1371/journal.pone.0254093 PMC8263274

[15] Chen KB Huang Y Jin XL. Electroacupuncture or transcutaneous electroacupuncture for postoperative ileus after abdominal surgery: a systematic review and meta-analysis. Int J Surg. 2019;70:93–101. 31494334 10.1016/j.ijsu.2019.08.034

[16] Xiong J Li H Li X. Electroacupuncture for postoperative pain management after total knee arthroplasty: protocol for a systematic review and meta-analysis. Medicine. 2018;97:e0014. 29489645 10.1097/MD.0000000000010014 PMC5851748

[17] Chen W Chen Z Li J. Electroacupuncture as an adjuvant approach to rehabilitation during postacute phase after total knee arthroplasty: a systematic review and meta-analysis of randomized controlled trials. Evid Based Complement Alternat Med. 2021;2021:9927699. 34394395 10.1155/2021/9927699 PMC8355970

[18] Li X Yu W Li H. Prospective, single-center comparison of transcranial direct current stimulation plus electroacupuncture and standard analgesia in patients after total knee arthroplasty: effect on rehabilitation and functional recovery. Med Sci Monit. 2021;27:e930363. 34103464 10.12659/MSM.930363 PMC8202124

[19] Jadad AR Moore RA Carroll D. Assessing the quality of reports of randomized clinical trials: is blinding necessary? Control Clin Trials. 1996;17:1–12. 8721797 10.1016/0197-2456(95)00134-4

[20] Song YY Ni GX. [Effect of preoperative intervention of electroacupuncture at Baliao point on postoperative complications of procedure for prolapsed and hemorrhoids]. Zhongguo Zhen Jiu. 2019;39:253–6. 30942010 10.13703/j.0255-2930.2019.03.008

[21] Wen Y Li J Long Q. [Electroacupuncture combined with catgut implantation for postoperative pain of mixed hemorrhoids]. Zhongguo Zhen Jiu. 2017;37:243–6. 29231428 10.13703/j.0255-2930.2017.03.006

[22] Long Q Li Y Li J. [Clinical observation of electroacupuncture combined with auricular point sticking therapy for anal pain of mixed hemorrhoid after external excision and internal ligation]. Zhongguo Zhen Jiu. 2018;38:580–5. 29971998 10.13703/j.0255-2930.2018.06.003

[23] Wu J Zhao Y Yang CM. [Effects of electroacupuncture preemptive intervention on postoperative pain of mixed hemorrhoids]. Zhongguo Zhen Jiu. 2014;34:279–83. 24843974

[24] Sun PL Yang W Zhang LC. [Effect of electroacupuncture at Chengshan (BL 57) on postoperative pain of mixed hemorrhoids]. Zhongguo Zhen Jiu. 2011;31:413–5. 21692285

[25] Xu J Yu W. Standardized clinical application and management of narcotic drugs and psychotropic substances. Beijing: People’s Health Publishing House. 2007.

[26] Li N He HB Wang CW. [Observation on therapeutic effect of electroacupuncture at Chengshan (BL 57) and Changqiang (GV 1) on hemorrhoidal pain]. Zhongguo Zhen Jiu. 2008;28:792–4. 19055280

[27] Zhang S Sun Y Li Q. Efficacy of Jinxuan hemorrhoid fumigation San with ear acupressure in the treatment of postoperative pain of mixed hemorrhoids. West China Med. 2015;30:1500–2.

[28] Wang L Jin X. Re-conceptualization of the eight super points. J Nanjing Univ Tradit Chin Med. 2014;30:4–7.

[29] Song X Tian S Zhang X. Positioning of the eight acupoints and their application in the treatment of adolescent dysmenorrhea with tui na. Massage Guiding. 2003;20:48.

[30] Jin X Ding SQ Shi FY. [Research on the angle and effective depth of deep acupuncture at baliao points by three-dimensional reconstruction of computed tomography]. Zhen Ci Yan Jiu. 2017;42:537–41. 29318863 10.13702/j.1000-0607.2017.06.014

[31] Zhao X Pang H. Clinical efficacy of Chengshan acupoint Journal of Beijing College of Acupuncture, Moxibustion and Bone Injury. 1998;5:28–9.

[32] Xu S Zhang H Gu Y. [Acupuncture from Tiaokou (ST 38) to Chengshan (BL 57) combined with local exercise for periarthritis]. Zhongguo Zhen Jiu. 2018;38:815–8. 30141290 10.13703/j.0255-2930.2018.08.005

[33] Higgins JPT Thomas J Chandler J. Cochrane Handbook for Systematic Reviews of Interventions version 6.1. London: Cochrane Collaboration. 2020.

